# Understanding patient and clinician perspectives of antibiotic use for the treatment of UTIs

**DOI:** 10.1093/geroni/igaa057.3363

**Published:** 2020-12-16

**Authors:** Susan Hannum, Ja Alah-Ai Heughan, Ayse Gurses, Morgan Katz, Jonathan Zenilman

**Affiliations:** 1 Johns Hopkins Bloomberg School of Public Health, Baltimore, Maryland, United States; 2 Johns Hopkins School of Medicine, Baltimore, Maryland, United States

## Abstract

Background. Multidrug resistant organisms are highly prevalent in post-acute long-term care [LTC] and skilled nursing facilities [SNF], driven by overdiagnosis of urinary tract infections [UTI] and overuse of antibiotics, despite clinical guidelines for UTI management. Using the Systems Engineering Initiative for Patient Safety [SEIPS] framework to understand sociotechnical work systems within LTC/SNFs, we are conducting mixed methodological research to examine work systems gaps that may require structural modification to ensure appropriate prescribing behaviors. Methods. To begin this research, we conducted semi-structured interviews with residents, caregivers, and clinical staff at three LTC/SNF locations. Resident and caregiver interviews queried knowledge, attitudes, and beliefs about UTIs and antibiotics, previous use, and communication with clinical staff. Clinical staff interviews queried procedures for diagnosing UTIs, prescribing decisions, communication with residents/caregivers, and resident/caregiver demand. Findings. Resident/caregiver interviews highlighted three common themes: (1) doctors have the right to deny antibiotics, but communication about decisions is critical; (2) trust doctors’ knowledge and use of objective testing for decision-making; (3) want detailed explanations and education about antibiotics, including potential side effects. Clinical staff described: (1) caregiver as the primary barrier, even with education about antibiotics; (2) using a general protocol for diagnosis, but also prior knowledge and experience with the resident; (3) importance of educating and communicating with residents/caregivers about antibiotic treatment, prescribing recommendations, or side effects. Conclusions. Our study highlights a gap in communication and workflow between residents, caregivers, and clinical staff that may be amendable to improved interventions that decrease inappropriate prescribing of antibiotics for this population.

